# Increased olfactory bulb acetylcholine bi-directionally modulates glomerular odor sensitivity

**DOI:** 10.1038/srep25808

**Published:** 2016-05-11

**Authors:** Mounir Bendahmane, M. Cameron Ogg, Matthew Ennis, Max L. Fletcher

**Affiliations:** 1 Department of Anatomy and Neurobiology, University of Tennessee Health Science Center, Memphis, TN 38163, USA.

## Abstract

The glomerular layer of the olfactory bulb (OB) receives heavy cholinergic input from the horizontal limb of the diagonal band of Broca (HDB) and expresses both muscarinic and nicotinic acetylcholine (ACh) receptors. However, the effects of ACh on OB glomerular odor responses remain unknown. Using calcium imaging in transgenic mice expressing the calcium indicator GCaMP2 in the mitral/tufted cells, we investigated the effect of ACh on the glomerular responses to increasing odor concentrations. Using HDB electrical stimulation and *in vivo* pharmacology, we find that increased OB ACh leads to dynamic, activity-dependent bi-directional modulation of glomerular odor response due to the combinatorial effects of both muscarinic and nicotinic activation. Using pharmacological manipulation to reveal the individual receptor type contributions, we find that m2 muscarinic receptor activation increases glomerular sensitivity to weak odor input whereas nicotinic receptor activation decreases sensitivity to strong input. Overall, we found that ACh in the OB increases glomerular sensitivity to odors and decreases activation thresholds. This effect, along with the decreased responses to strong odor input, reduces the response intensity range of individual glomeruli to increasing concentration making them more similar across the entire concentration range. As a result, odor representations are more similar as concentration increases.

Odors are detected by olfactory sensory neurons (OSNs) in the nasal cavity that express a single receptor type. OSNs project their axons into specific glomeruli in the olfactory bulb (OB) where they form excitatory synapses onto a complex circuit of interneurons and mitral/tufted (M/T) cells. This convergence forms the basis of the glomerular odor map whereby odor information is represented by distinct spatio-temporal patterns of M/T cell apical dendrite glomerular activity.

Cholinergic innervation of the OB arises from the horizontal limb of the diagonal band of Broca (HDB)^1^. These fibers terminate densely in the glomerular layer and moderately in the sub-glomerular layers. This projection pattern is paralleled by expression of muscarinic and nicotinic ACh receptor (AChR) subtypes^2^,^3^,^4^,^5^,^6^,^7^,^8^. ACh release by the basal forebrain cholinergic system has been demonstrated to be involved in arousal, attention, and learning. During active, awake states, cholinergic neurons display increased activity^9^,^10^ and are active during odor investigation and learning^11^. Similarly, cortical ACh release is increased by novel sensory stimuli^12^,^13^ and by arousing or aversive events^14^,^15^. ACh release is hypothesized to have several effects including cue detection, enhancing sensory coding of salient stimuli, and facilitating memory encoding^16^,^17^.

Previous studies have demonstrated that ACh release and activation of AChRs facilitate olfactory learning, memory, odor discrimination, and generalization^18^,^19^,^20^,^21^,^22^,^23^,^24^. However, the mechanisms by which ACh release facilitates these behaviors are not understood, especially in terms of OB odor processing. Previous *in vitro* electrophysiology studies have shown that ACh or cholinergic agonists can exert excitatory or inhibitory effects that depend on cell (M/T vs. inhibitory interneurons) and AChR subtype3,^6^,^7^,^22^,^25^,^26^. How these varying cellular effects impact odor responses has been less well studied. More recent *in vivo* studies using optogenetic approaches have demonstrated that activation of HDB ACh neurons or ACh fibers in the OB can lead to both increases and decreases in M/T cell odor responses^26^,^27^.

However, several questions remain regarding the function of ACh modulation of OB odor processing, especially in terms of glomerular odor representation. The glomerular layer is the most heavily targeted by HDB ACh input^28^ and contains cholinoreceptive cell types expressing muscarinic (mAChR) and nicotinic (nAChR) receptors^2^,^29^. Despite this, it is still unknown if and how synaptically-released ACh modulates M/T cell glomerular odor responses to OSN input, and if potential ACh actions vary with odor intensity and the AChR types involved.

Here, we used transgenic mice expressing the calcium indicator GCaMP2 in OB M/T cells^30^ to investigate cholinergic modulation of M/T cell glomerular odor representations *in vivo*. Overall, we find that increased OB ACh release via HDB stimulation or pharmacological activation leads to dynamic, activity-dependent modulation of glomerular odor sensitivity, resulting in increased individual glomerular sensitivity to weaker odor input and decreased sensitivity to stronger odor input. Further, through the use of direct OB pharmacological manipulation, we demonstrate that the increased sensitivity is due to m2R activation, while the decreased sensitivity is due to nicotinic receptor activation. The interplay between these two receptor types leads to more similar overall glomerular representations of a given odorant across a large range of odor concentrations.

## Results

### HDB activation modulates glomerular odor responses

**Figure 1:**
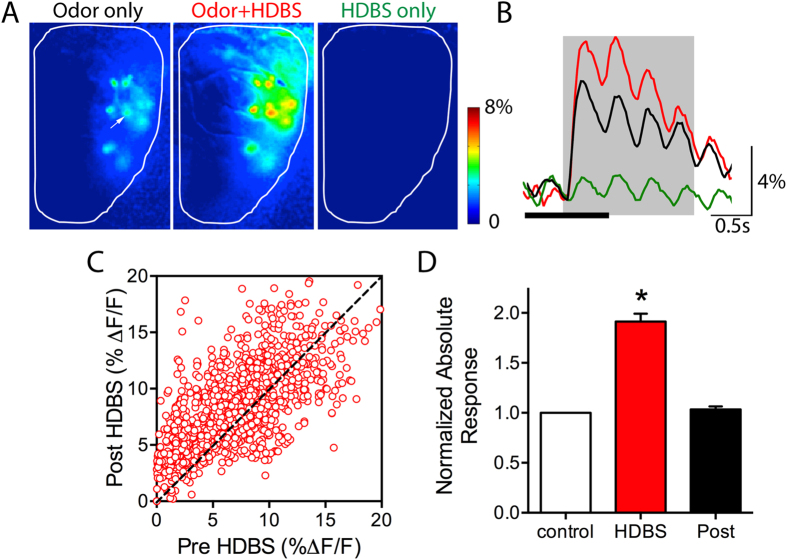
HDBS affects glomerular odor responses. (**A**) Glomerular responses recorded from the dorsal surface of the OB in a GCaMP2 expressing mouse. Odor responses to 1% methyl valerate are increased when the odorant is paired with HDBS while HDBS alone with no odorant does not induce any response. (**B**) Example traces after photo-bleaching correction of the glomerulus indicated by the arrow in A, in the control, HDBS + odor, and HDBS alone conditions. The oscillations reflect the respiration cycle; black bar at bottom indicates time of HDBS. (**C**) Plot of the HDBS response modulation as function of the control responses (1236 pre-post HDBS paired responses). Odor responses are both above and below the dashed unity gain line, showing both increased and decreased odor response magnitude by HDBS. Most decreases are observed when the control response magnitudes are relatively high (above 5% Δf/f) and observed more frequently as the control responses increase. (**D**) Bar graphs showing the mean absolute odorant response magnitude change (91.3 ± 7.96% increase) induced by HDBS compared to the pre-HDBS (Control); responses return to control levels two minutes following HDBS (Post). *p < 0.01.

Using wide field optical calcium imaging, we recorded glomerular odor responses to increasing concentrations of odorants from anesthetized transgenic GCaMP2 animals. We investigated the effect of HDBS on odor responses from 230 glomeruli in 16 mice. HDBS did not appear to change respiratory frequency and HDBS effects were observed in all responsive glomeruli regardless of their location on the dorsal surface of the OB. We first assessed the effect of HDBS on baseline glomerular activity in the absence of odor input, finding no differences in glomerular fluorescence intensity before and during HDBS (paired t-test: t = 1.047, df = 27 p = 0.30) (Fig. 1A,B). To determine whether the HDBS effect is long lasting, in a subset of glomeruli, odor responses were measured prior to HDBS (pre-), paired with HDBS, and again 2 minutes following HDBS (post-) (n = 11 mice, 199 glomeruli). One-way ANOVA analysis showed a significant difference between the three conditions (one way ANOVA: F (2, 1488)  =  111.9, p < 0.001). Bonferroni’s multiple comparison tests showed that HDBS induced a significant 91.3 ± 7.9% absolute change relative to control in glomerular odor responses compared to pre-HDBS responses (Fig. 1C,D). However, odor responses within two minutes following HDBS were not different than pre-HDBS responses (3.4 ± 2.9% change compared to control), suggesting that HDBS effects were transient and had no lasting effects on odor responses (Fig. 1D). In order to determine the net effect of HDBS, for all animals and glomeruli, we averaged odor responses for all the pre- trials and compared them to the corresponding averaged HDBS odor responses (n = 16 mice, 230 glomeruli, 1236 pre-post HDBS pairings). Overall, we found that HDBS delivered during odor trials leads to a significant mean absolute change of 2.67 ± 0.06% ΔF/F (paired t-test t = 32.41, df = 1235, p < 0.001) compared to control responses. As reported previously in M cells^27^, we found HDBS could lead to either increases or decreases in the magnitude of individual glomerular responses to the different odors. HDBS-evoked responses were plotted as a function of their pre-HDBS response. The distribution showed both enhanced (above the gain line) and reduced (below the gain line) odor responses following HDBS (Fig. 1C).

### HDB stimulation bidirectionally affects individual glomerular odor responses depending on relative odor intensity

**Figure 2:**
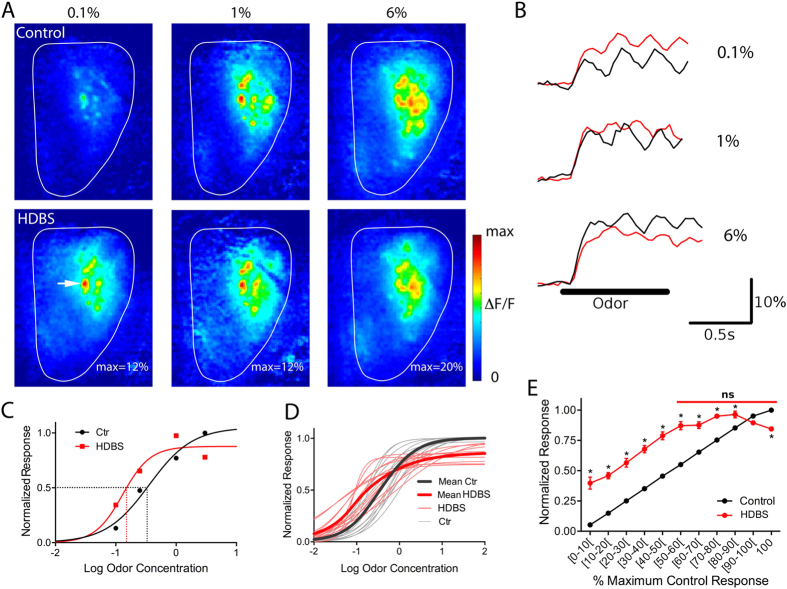
HDBS bidirectionally modulates glomerular odor responses. (**A**) Responses to increasing concentrations of methyl valerate in control (top) and HDBS (bottom) conditions. Glomerular odor responses are increased by HDBS for the low and middle range concentrations while they are decreased at the highest concentration. (**B**) Fluorescence signal traces for the glomerulus indicated by arrow in A. Response intensity is increased for the low and middle range concentration while it is decreased for the high concentration. (**C**) Example of a sigmoid fit of Log odor concentration (Log 1% = 0) - normalized odor responses curves for a single glomerulus in control and HDBS conditions. HDBS shifts the curve to the left resulting on decreasing the EC50 (dashed line) and decreases the maximum response. (**D**) Sigmoid fit of Log odor concentration – normalized odor responses of all the glomeruli shown in A, the thick lines show the average curves in both control (black) and HDBS (red) conditions. The grey and red curves are individual curves of each responsive glomerulus. (**E**) Population normalized control and HDBS curves, plotted as function of the normalized control responses divided into 10% intervals. The curves show that odor responses are increased by HDBS from the lowest concentration to 80%, the HDBS curve crosses the control curve in the [80–90%] interval and the highest responses are significantly decreased after HDBS. (Mean ± SEM, *: p < 0.01). Red bar: HDBS responses are not significant from each other within the [50–100%] interval.

The distribution of HDBS responses as a function of the control responses showed that reductions in odor responses following HDBS occur more often with stronger glomerular control responses (Fig. 1C). This suggested a possible relationship between the strength of the control response and the effect of HDBS, where the weaker responses are enhanced and the strong ones reduced. However, responses to the same odorant at the same concentration may be increased by HDBS in some glomeruli and decreased in others, suggesting that the relationship occurs relative to individual glomerular responses rather than the absolute intensity of the odor stimulus. To investigate this, the effects of HDBS on individual glomeruli were tested across a large range of odorant concentrations. This revealed that HDBS enhanced responses to low odorant concentrations and suppressed responses to high odorant concentrations (Fig. 2A,B). To further address this relationship, we normalized the control and HDBS-evoked responses for every glomerulus to its maximum response in control conditions, and plotted them as function of Log odor concentration (Fig. 2C,D). We then compared individual odor-concentration-response curves in both control and HDBS conditions in 188 glomeruli obtained from 15 mice (972 pre-post HDBS pairings). This revealed that odor responses to relatively weak and moderate odor concentrations were increased, while vigorous responses evoked by near maximal concentrations were reduced in individual glomeruli (Fig. 2C,D).

Differences in individual glomerular sensitivity to a given odorant within and between animals, i.e. a given concentration induces a weak response in some glomeruli and a strong response in others, can make it difficult to extract mean population effects. Therefore, we pooled the control and HDBS normalized responses and plotted the average control and the corresponding HDBS normalized responses as a function of normalized control maximal response for each glomerulus binned in 10%-amplitude intervals (Fig. 2E). One-way ANOVA showed differences between control and HDBS normalized responses across all intervals (F(21, 1600) = 203.2, p < 0.0001); post hoc Bonferroni’s multiple comparison tests were then used to compare the control and the corresponding HDBS responses for each interval. As expected, the average normalized control yielded a linear plot of increasing responses towards the normalized maximum for all glomeruli. The average normalized HDBS response curve exhibited a significant shift upward reflecting enhanced responses for all intervals below 80% of the maximum response) with increases ranging from 21.2 ± 1.8% to 34.0 ± 2.8%. Above the 80% interval, the HDBS curve intersects the control curve (i.e., no response change) followed by a significant shift downward, with a 15.8 ± 1.5% decrease of the average normalized maximum response (100%). Together, these results indicate that ACh release enhances odor response over much of the dynamic range of an individual glomerulus, with suppression occurring at relatively high odor concentrations that drive strong responses approaching the saturation limit.

### Pharmacologically increasing ACh in the OB mimics HDBS effects on glomerular sensitivity

**Figure 3:**
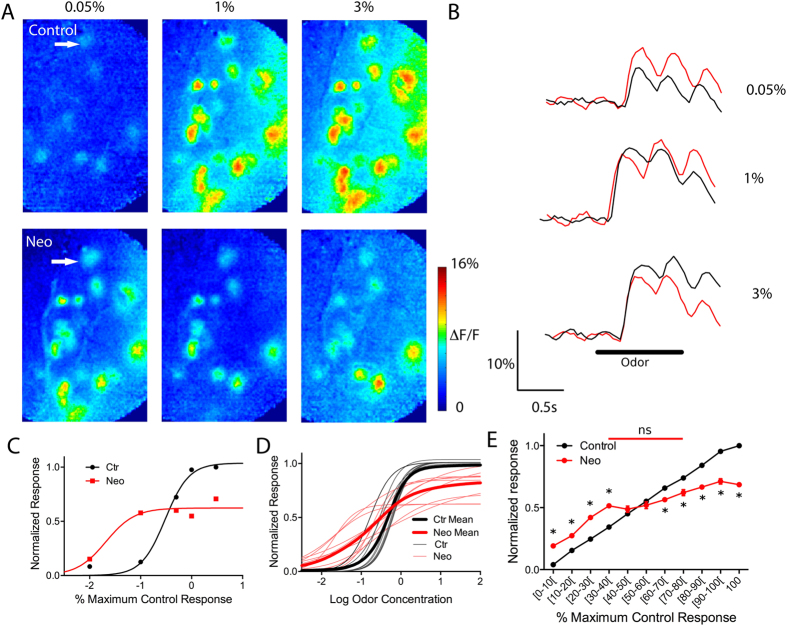
Neostigmine bidirectionally modulates glomerular odor responses. (**A**) 10× magnification images of responses to increasing concentrations of 2 heptanone in control condition (top) and during neostigmine (Neo) application (bottom). Glomerular odor responses are increased after neostigmine application for the low concentration, they remain unchanged at the middle range concentration and are decreased at the high concentration. (**B**) Fluorescence signal traces for the selected glomerulus shown in A showing response increase (for all the 0.05% concentration), no change (1.0%) or decrease (3%). (**C**) Example of a sigmoid fit of Log odor concentration- normalized odor responses curves in control and during neostigmine application. Neostigmine shifts the curve to the left resulting on decreasing the EC50 and also decreases the maximum response. (**D**) Sigmoid fit of Log odor concentration – normalized odor responses of all the glomeruli shown in A, the thick lines show the average curves in both control (black) and neostigmine (red) conditions. The grey and red curves are individual curves of each responsive glomerulus before and during neostigmine application. (**E**) Population normalized control and post-neostigmine curves, plotted as function of the normalized control responses divided into 10% intervals. The curves show that odor responses are increased by neostigmine from the [0–40%] intervals, are unchanged around 50% causing the post-neostigmine curve to cross the control curve at this point, and responses are significantly decreased in the [60–100%] intervals. *p < 0.01, ns: p > 0.05). Red bar: post-neostigmine responses are not significant from each other within the [30–80%[interval.

The HDB contains both cholinergic and GABAergic neurons that project to multiple regions^1^. Therefore, it is possible that the HDBS-evoked effects on glomerular odor responses could be due to either ACh and/or GABA receptor activation. Moreover, an indirect effect of ACh release in other olfactory regions that provide centrifugal projections to the OB is also a possibility. To isolate OB cholinergic effects, we used OB bath application of the cholinesterase inhibitor neostigmine to specifically increase ACh. Cholinesterase blockers rapidly increase endogenous ACh levels^31^ and are known to have strong effects on odor discrimination and OB single neuron activity^19^,^21^,^22^. In nine mice, we recorded glomerular odor responses from 197 glomeruli to different odors and concentrations before and after OB topical application of neostigmine. Similar to HDBS, neostigmine induced an absolute increase of 2.28 ± 0.04% ΔF/F in the glomerular odor-evoked responses compared to control trials (1095 pre-post neostigmine paired responses, paired t-test, t = 31.65, df = 1094, p < 0.001); Fig. 3A,B.

Using the same method as for HDBS, we selected glomeruli that displayed complete response-concentration curves in the control condition and plotted individual pre- and post-neostigmine responses normalized to maximum pre-neostigmine responses (6 animals, 79 glomeruli, 674 pre-post neostigmine pairings). As observed with HDBS, weak-to-moderate responses were enhanced by neostigmine application while strong responses were reduced (Fig. 3C,D). For these selected glomeruli, we plotted the average pre-neostigmine and the corresponding post-neostigmine normalized responses as a function of normalized pre-neostigmine responses binned in 10%-amplitude intervals (Fig. 3E). The normalized pre-neostigmine response yielded a linear plot of increasing response towards the normalized maximum for all glomeruli, with each bin step significantly different from the previous (ANOVA: F(10, 672) = 12440, p < 0.0001; Bonferroni’s multiple comparison tests). Following neostigmine application, the normalized plot showed a significant shift upward in responses below 40% of the maximum response and a significant shift downward in responses above 60% of the maximum response (Fig. 3E). Despite the fact that the post-neostigmine curve crosses the control curve at a lower point than does the post-HDBS curve, bidirectional odor response modulation is also observed with neostigmine.

### The dual cholinergic effect on glomerular responses is due to activation of both muscarinic and nicotinic receptors

**Figure 4:**
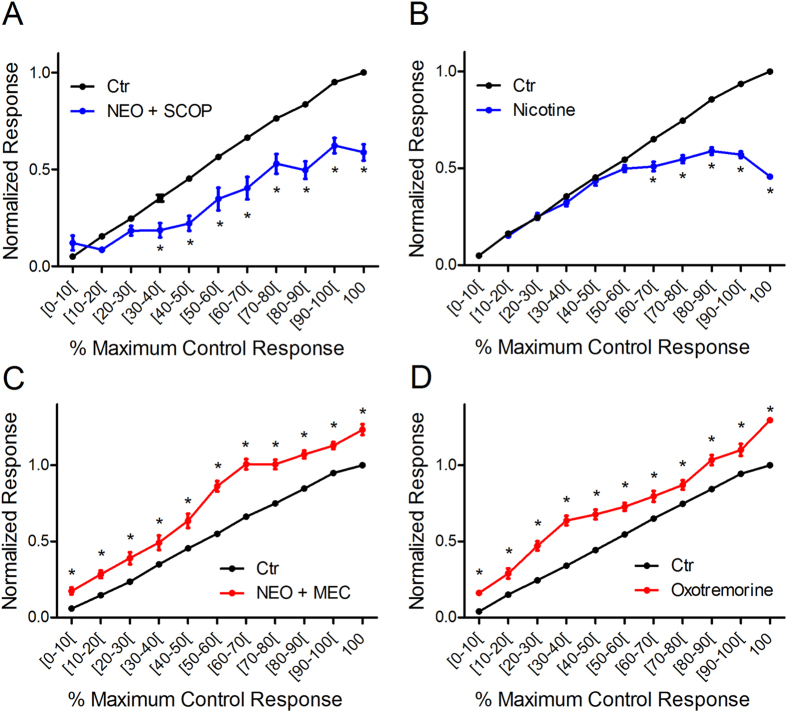
Glomerular odor responses are increased and decreased by muscarinic and by nicotinic receptor activation. (**A**) Nicotinic receptor activation using neostigmine bath application combined with the mAChR antagonist scopolamine did not change weak odor responses and decreased odor responses above [30–40%] interval of the maximum control response. (**B**) The muscarinic receptor agonist oxotremorine increased odor responses at all intervals. (**C**) Nicotine did not change low to moderate odor responses and decreased odor responses above 60% of maximum control response. (**D**) Similar to oxotremorine, neostigmine in the presence of the nicotinic receptor antagonist mecamylamine increased all odor responses across the entire response range. *p < 0.01.

As the preceding data suggest that both HDBS and increased OB ACh lead to bidirectional shifts in glomerular response patterns as a function of odor concentration, we next investigated the receptor basis of this modulation via pharmacological manipulations. We first investigated the potential role of nAChR activation. We co-applied neostigmine with the mAChR antagonist scopolamine to the OB (n = 3 mice, 36 glomeruli, 185 pre-post-application paired responses) and compared odor responses pre- and post-application normalized to maximum pre-application responses as above (Fig. 4A) to reveal any nicotinic component. Compared to control, blocking mAChRs in the presence of increased synaptic ACh induced significant differences in mean normalized responses across intervals (one-way ANOVA f(21, 369) = 106.0). Post hoc Bonferroni’s multiple comparison tests showed that in the presence of scopolamine, neostigmine lead to significant decreases across a large portion of the intervals (≥30% of the maximum control responses) (Fig. 4A) suggesting that nAChR activation leads to decreased odor responses. To further confirm this, we applied nicotine, a general nAChR agonist, and measured odor responses following the same protocol (n = 3 animals, 44 glomeruli, 296 pre-post-nicotine pairings). The post-nicotine responses also showed significant decreases from control albeit at higher relative intensities (≥60% of the maximum control responses) (one-way ANOVA F(19,572) = 367; p < 0.001, post hoc Bonferonni multiple comparison tests) (Fig. 4B). Further analysis revealed that nAChR-induced decreases are larger as intensity increases. nAChR activation via neostigmine + scopolamine induced a larger change at the highest normalized response compared to the lowest normalized response (change at the lowest (7.0 ± 3.8%) versus highest (42.2 ± 3.5%) normalized responses, t-test: t = 7.11, df = 44, p < 0.001). Similarly, nAChR activation via nicotine application also induced a larger change at the highest normalized response compared to the lowest normalized response (change at the lowest (1.0 ± 1.4%) versus highest (54.2 ± 1.3%) normalized responses, t-test: t = 26.26, df = 101, p < 0.001).

To explore the role of mAChR activation, we first co-applied neostigmine with the nAChR antagonist mecamylamine to the OB (n = 3 mice, 48 glomeruli, 204 pre-post pairings). In the presence of mecamylamine, neostigmine enhanced odor responses across all concentrations, and failed to reduce responses at any intensity (ANOVA F(21,408) = 210.1, post hoc Bonferroni’s multiple comparison tests) (Fig. 4C). To further confirm this, we bath applied the general mAChR agonist oxotremorine (10 μM) in another group of mice (n = 6) and measured odor responses from 102 glomeruli, (522 pre-post-oxotremorine pairings). OB oxotremorine application enhanced responses at all odorant concentrations (Fig. 4D) (ANOVA F(21,1052) = 301.6, post hoc Bonferroni’s multiple comparison tests). Further analysis of the effects of mAChR activation, either through oxotremorine or neostigmine + mecamylamine application, reveals that mAChR-induced increases lead to a linear shift in the response line, with a slope near 1 (1.01 ± 0.05 for oxotremorine and 1.10 ± 0.05 for neostigmine + mecamylamine) suggesting a gain increase across all concentrations.

These data suggest that nAChR activation alone leads to an activity dependent decrease in glomerular odor responses while mAChR activation alone leads to a uniform increase in the glomerular odor responses across all response intensities. Taken together, the data to this point suggest that the bidirectional shifts in glomerular odor responses are due to the activation of both mAChRs and nAChRs.

### Muscarinic-induced increases in glomerular sensitivity are due to m2R activation

**Figure 5:**
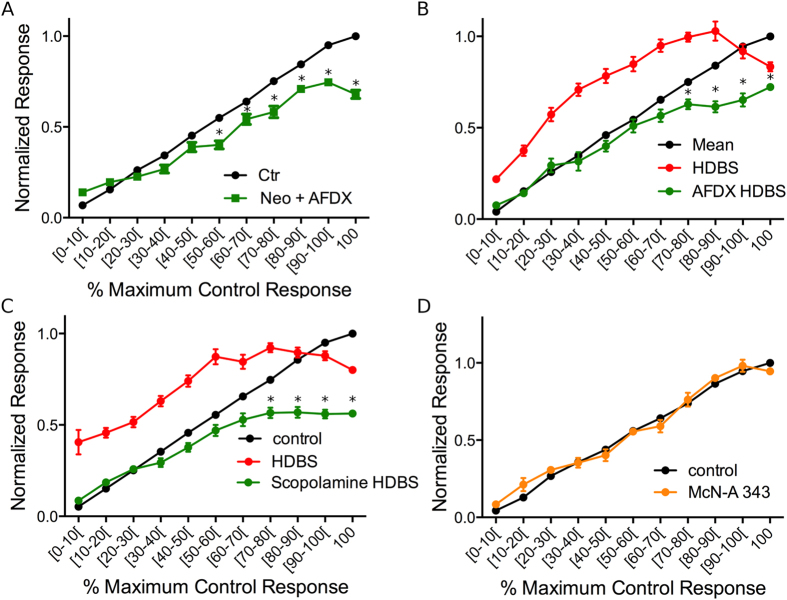
M2R activation increases glomerular responses. (**A**) Neostigmine failed to increase glomerular odor responses in the presence of the M2R antagonist AF-DX 116; also note that decreased responses are observed above 30% of the maximum control response. (**B**) HDBS increased odor responses in the [0–90%] intervals, and decreased responses at 100%. The M2R antagonist AF-DX 116 blocks the response facilitation and transforms responses above the [60–70%] interval to inhibition. (**C**) As in (B), the HDBS enhancement of odor responses is blocked and converted to response suppression by the general mAChR blocker scopolamine above the [30–40%] interval. **D.** The M1R agonist McN-A 343 has no effect on HDBS-evoked glomerular odor response modulation. *p < 0.01 compared to control condition.

Previous *in vitro* studies reported that mAChR activation suppresses PG cell activity, potentially through the m2R AChR subtype^25^,^32^. This mechanism has been proposed to enhance M/T cell responses to odors via reduced inhibition^25^,^32^. Based on this, we tested whether the muscarinic-induced increase in glomerular response is mediated by m2R activation via bath application of neostigmine in the presence of AF-DX116, an m2R-specific antagonist (n = 5 animals, 67 glomeruli, 358 pre-post pairings). Overall, the mean responses between intervals in the pre- and post- application conditions were significantly different (one-way ANOVA F(21,652) = 216.3, p < 0.001). Post-hoc tests showed that in the presence of AF-DX116, neostigmine failed to increase odor responses. However, response suppression, likely mediated via nAChR activation, was observed at responses at and above 50% of the maximum control responses (Fig. 4A). Further, a comparison of post-neostigmine + AF-DX116 responses and post- neostigmine + scopolamine responses showed no differences between scopolamine or AFDX in the range of responses that were increased by neostigmine application alone (0–40%) (one-way ANOVA (F(21,500) = 47.09, p < 0.001). To further investigate the role of m2R, we tested the HDBS protocol before and after OB application of AF-DX116 in another group of mice (n = 4, 54 glomeruli, 286 pre- post- AF-DX116 paired responses). As above, HDBS bidirectionaly modulated responses as a function of odorant concentration. However, AF-DX116 completely blocked the HDBS enhancement of responses (Fig. 5B). The data also show that HDBS failed to enhance odor responses in the presence of AF-DX116, while significant decreases above 60% of the maximum control responses were still observed (one-way ANOVA F (32,897) = 140.4, p < 0.001, Bonferroni multiple comparison tests).

To confirm that the muscarinic-induced odor response increase is completely attributable to m2Rs, we next compared m2R blockade to general muscarinic receptor blockade with scopolamine using the preceding HDBS testing protocol. The control, HDBS, and HDBS + scopolamine normalized average response curves were significantly different (one-way ANOVA F(32, 1578) = 127.6; n = 7 animals, 109 glomeruli, 538 pre-post pairs). In the presence of scopolamine, HDBS failed to enhance odor responses while a significant decrease was observed for the intervals that evoked responses above 60% of the maximum (post hoc Bonferronni’s multiple comparison tests) (Fig. 5C). Moreover, as observed with neostigmine, no significant difference was observed between HDBS + AF-DX116 and HDBS + scopolamine in the response range where HDBS is expected to enhance odor responses (0–80% of maximum response; one-way ANOVA f (21,825) = 42.99, p < 0.001, Bonferroni’s multiple comparison tests).

This suggests that the m2R subtype plays a major role in the muscarinic-induced enhancement of glomerular odor responses. However, m1Rs are also present in the OB and have been demonstrated to play a role in deeper, mitral cell-granule cell interactions^6^,^7^,^33^. To explore the potential role of m1Rs, we applied the m1R-specific agonist McN-A343 alone and measured pre- and post- application odor responses (n = 3 animals, 32 glomeruli, 134 pre-post pairings). One-way ANOVA analysis showed significant differences between the different mean responses across intervals in both control and McN-A343 conditions (F(21,246) = 228.1, p < 0.001). However, post hoc tests showed no differences in corresponding response magnitude intervals between the control and McN-A343 curves (Fig. 5D). At the end of the experiment, 1% lidocaine application completely blocked odor responses, showing effective drug penetration in these experiments. Together, these results suggest that the increases in glomerular responses evoked by ACh release in the OB are a result of m2R activation.

### Functional implications: ACh increases efficient concentration range and leads to more similar representations of odor concentrations

The results to this point indicate that AChR activation increases weak-to-moderate and decreases strong odor responses. Two potential consequences could result from this dual effect: **1.** By increasing weak responses, increased OB ACh can expand the range of odor concentrations that induce glomerular responses by lowering the activation threshold. **2.** By decreasing the intensity of maximum odor responses, ACh could reduce the total magnitude of the responses’ intensity range. This hypothesis implies that HDBS may induce variation in two parameters, the odor concentration range that induces a measurable response and the response intensity. Because these parameters may be dependent on each other, when studying one parameter, we fixed the other parameter by normalizing the responses to the maximum response of either each condition (pre- and HDBS) or only control (pre-) condition (see Methods).

**Figure 6:**
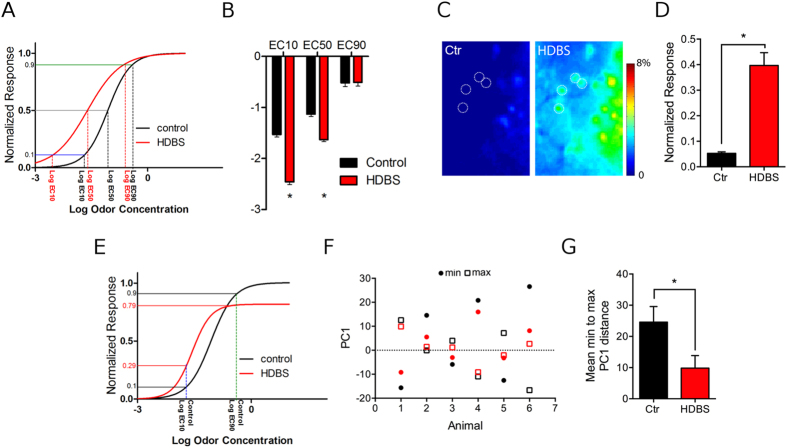
Functional implications of HDBS on unitary and population glomerular odor coding. (**A**) Log concentration–response curves in control and HDBS conditions. Odor responses are normalized to every condition’s maximum response. Log EC10, 50 and 90 are determined by the projection on the x axis of the crossing point of the curves with the blue (y = 0.1) grey (y = 0.5) and the green (y = 0.9) lines respectively. Log EC10 and 50 are shifted to the left by HDBS (red) compared to control (black), while Log EC90 is similar for both conditions. (**B**) Average Log EC10, 50 and 90 in control and HDBS conditions. HDBS significantly decreases Log EC10 by 0.93 Log unit and Log EC50 by 0.5 Log unit while not affecting EC90. (**C**) Responses of the dorsal OB (4× magnification) surface to 0.1% methyl valerate in control and HDBS conditions. HDBS increases the odor responses of the glomeruli activated in control condition and induced odor responses in additional glomeruli (dashed circles) that did not respond in the control condition. (**D**) Average mean response of the glomeruli that showed below threshold relative ΔF/F. HDBS increases the mean ΔF/F of these glomeruli from 5.2 ± 0.5% in control condition to 39.7 ± 4.9% after HDBS. (**E**) Log concentration–response curves in control and HDBS conditions for the same glomerulus as in A. Odor responses are normalized to control maximum response. Normalized responses to the Log concentrations EC10 and 90 were compared in both control and HDBS conditions, determined by the projection of the y axis of the crossing point of the curves with the lines x = control EC10 and x = control EC90 respectively. The distance between the Log EC10 and Log EC90 responses is smaller in HDBS condition [0.29–0.79] than in control condition [10–90]. (**F**) Graphic representation of the projections of minimum (circles) and maximum (squares) concentration on the principal component analysis on PC1 for the six selected animals. The distance from minimum to maximum is bigger in control condition (black) than with HDBS (red). (**G**) Mean PC1 minimum to maximum distances decreased by HDBS (n = 6), *p < 0.01.

To investigate the first parameter, i.e. the effect of HDBS on the concentration range that elicits measurable glomerular responses, for each glomerulus, we fixed the response intensity parameter by normalizing the experimentally obtained odor responses to the maximum response for each condition. We obtained a full response range from 0 to 100% for each condition, which allowed us to assess the effects of HDBS on the concentration range in every condition regardless of its effect on induced response intensity. We fit the normalized responses to sigmoid curves as function of Log odor concentration as previously described in control and HDBS conditions (Fig. 6A). Then, we compared the Log concentrations that elicited 10%, 50% and 90% of the maximum response for each condition (respectively Log EC10, Log EC50 and Log EC90) (Fig. 6B). As expected, HDBS significantly shifted the Log EC10 by 0.93 Log units towards lower concentrations (control Log EC10: −1.53 ± 0.05; HDBS Log EC10: −2.46 ± 0.05, df = 103, t = 16.46, p < 0.001). A smaller shift of the Log EC50 (by 0.5 Log units) was induced by HDBS (control LogEC50: −1.13 ± 0.05; HDBS LogEC50: −1.63 ± 0.04, t = 14.67, df = 103, n = 104, p < 0.001). However, the Log EC90 was not affected by HDBS (control Log EC90: −0.52 ± 0.07; HDBS Log EC90: −0.51 ± 0.07, df = 103, t = 0.14, p = 0.88). By shifting the EC10 towards a lower concentration and keeping the EC90 fixed, HDBS flattens the slope of the linear part of the curve (control: 2.38 ± 0.34, HDB: 1.15 ± 0.11, t = 3.51, df = 103, p < 0.001). Consequently, the concentration range that elicits responses between the lower and higher plateaus of the curve broadens^34^. These data indicate that HDBS decreases the theoretical odor concentration needed to elicit threshold responses but does not change the concentration that evokes the maximum responses, regardless of their absolute value. To experimentally verify this finding based on theoretical sigmoid fit, we identified a subset of glomeruli that displayed sub-threshold responses at weak odor concentrations under control conditions. We then compared their relative to maximum control ΔF/F at that concentration in control and HDBS conditions. In these glomeruli, HDBS significantly increased responses above threshold (control = 5.2 ± 0.5%, HDBS = 39.7 ± 4.9%, paired t-test, t = 6.83, df = 27, p < 0.001) (Fig. 6C,D). Overall, HDBS broadens the range of concentrations that elicit odor responses by increasing glomerular sensitivity to relatively weak odor input and decreasing glomerular activation threshold.

To investigate the second parameter, i.e. the effect of HDBS on response intensity, we studied the variation of responses normalized to control maximum response while fixing the concentrations to the concentration range between control Log EC10 and Log EC90. Theoretical projections based on the sigmoid fit show that the possible odor responses are constrained to a smaller interval after HDBS (Fig. 6E). To further confirm this finding and translate it to our experimental data, we compared the normalized minimum responses [0–10%[and the maximum possible response at 100% in the control condition to the corresponding normalized HDBS response. We found that HDBS increased the average response to concentrations that induced control responses in the [0–10%[interval (control = 5.2 ± 0.5%, HDBS = 39.7 ± 4.9%, paired t-test, t = 6.83, df = 27, p < 0.001), while the average response to the concentrations that induced maximum control responses was decreased (control = 100%, HDBS = 84.4 ± 1.6%, paired t-test, t = 9.36 , df = 161 , p < 0.001). Consequently, HDBS parses possible odor responses [0% – 100%] into a narrower interval range [39.7 ± 4.9% – 84.4 ± 1.6%], thus making the intensity of responses to lower and higher concentrations closer.

Finally, we asked how the HDBS-induced response changes at the individual glomerular level impact the broad population response for a given odorant. To test this, we selected six mice with activated glomeruli that fit pre- and post-HDBS curves according to our criteria (see Methods) and performed principal component analysis (PCA) on these glomeruli across the entire concentration range of ethyl tiglate. For each animal, the population response to each concentration is represented by point in principal component space. This analysis showed that the different concentrations were closer to each other in the principal component space after HDBS than in the control condition. To quantify this change in each animal, we compared the distance between the minimum and maximum concentration along PC1 (which accounted for >68% of total variation (Fig. 6F). This comparison showed that the average difference between the minimum and the maximum concentrations was smaller with HDBS than in the control condition (control 22.9 ± 4.5; HDBS: 10.3 ± 3.4, t = 2.96, df = 5, p < 0.03) (Fig. 6G). This suggests that a major role of OB AChR activation is to increase the similarity of glomerular responses across a broad concentration range.

## Discussion

We find that increased OB ACh leads to bidirectional, activity-dependent modulation of M/T cell glomerular odor responses whereby weak-to-moderate responses are enhanced and strong responses are reduced. This effect was observed regardless of odorant, glomerular spatial location or baseline glomerular sensitivity. Further, this enhancement is due to activation of m2R while reduction of nearly saturated responses with high odorant concentrations is due to nAChR activation. Together, these mechanisms serve to increase the similarity of glomerular odor representations across a wider concentration range.

The bidirectional modulation of individual glomerular response was similar whether HDBS or OB neostigmine application was used. These results, together with findings that OB AChR agonists and antagonists respectively mimicked or blocked the effects of HDBS, strongly suggest that the observed modulation is due to direct actions of ACh within the OB and not due to centrifugal influences from other brain regions affected by HDBS. Despite the presence of GABAergic HDB projection neurons, we found that HDBS effects on M/T cell glomerular odor response can be completely explained through cholinergic activation, leaving the role of HDB GABAergic projections unclear.

Based upon previous M/T cell glomerular calcium imaging studies^30^,^35^, the majority of the odor-evoked glomerular signal reflects activity in the distal dendrites of excitatory cells with little representation of deeper somatic activity. Thus, the changes reported here likely reflect modulation of M/T cell dendritic glomerular responses to OSN input and may not reflect cholinergic modulation at deeper OB layers. Our findings fit well with findings of both increased and decreased M/T somatic odor responses following optogenetic HDB stimulation^27^ and suggest that at least part of the somatic odor responses modulation is due to the cholinergic effects seen at the glomerular level. A more recent study using optogenetic stimulation of OB ACh fibers reported only response increases^26^. These differences could be due to location of stimulation (OB versus HDB), differences in stimulation protocols, or lack of high intensity odor stimulation where we observe the inhibitory effects.

Both nAChRs and mAChRs are present in the glomerular layer and both subtypes can influence output cells and interneurons^3^,^4^,^5^,^6^,^7^,^32^. Studies of cholinergic modulation of OB neurons have described contradictory effects of cholinergic modulation depending on whether the study was done *in vitro* or *in vivo* and whether the focus was on spontaneous or induced activity^4^,^5^,^6^,^7^,^25^,^26^,^27^,^36^,^37^. Our results are the first to describe how n- and mAChR work individually and together to modulate glomerular responses based on the intensity of the sensory input.

With nAChR activation, we found an intensity-dependent suppression of glomerular activity that decreased strong odor responses engaged by higher odorant concentrations. While the mechanism for this effect is not known, the fact that this supression is stronger at high intensities suggests that it is possibly due to feedback inhibtion. Nicotinic activation can induce glutamate release in the prefrontal cortex^38^ as well as the OB^4^. In the OB, increased glutamate release is thought to excite PG cells and increase feedback inhibition onto M/T cells resulting in decreased responses to OSN input^4^.

Interestingly, the nAChR-driven effect occurred at a lower concentration range with neostigmine than with HDBS or nicotinic agonists. Previous work has demonstrated that stronger basal forebrain stimulation causes stronger nAChR effects on cortical receptive field sharpening^39^ suggesting that nAChR activation is sensitive to ACh levels. The neostigmine concentration used here likely lead to maximal and tonically elevated OB ACh concentrations^31^, while brief HDBS produces a phasic increase in ACh. Consistent with this, odor responses returned to control values within minutes of HDBS while neostigmine effects persisted for over 60 minutes (data not shown). Higher ACh levels produced by neostigmine may explain why the nAChR-driven inhibition occurred at lower odor concentrations compared to the HDBS. It is noteworthy that neostigmine combined with the mAChR blocker scopolamine induced decreases of weaker responses compared to nicotine (10 μM) (Fig. 4A,B). This may be due to the concentration of nicotine used.

In contrast, mAChR activation leads to increased responses at all odor concentrations. This was observed following HDBS or neostigmine in combination with nAChR antagonists, as well as with a mAChR agonist. In all of these conditions, mAChR activation leads to a similar increase in response magnitude at all odor concentrations, suggesting that this effect is likely a gain shift. As both m1R and m2R are located in the OB^2^,^29^, we explored the potential role of these receptor types in mediating the increased responses observed. Overall, we found that application of the m2R antagonist AF-DX116 combined with neostigmine or HDBS completely blocks the response increase and leaves only the nAChR-mediated response suppression expected at higher intensities. Further, application of an m1R agonist alone had no effect on responses. Together, these results demonstrate that the increased glomerular responses are solely due to m2R activation.

mAChRs are expressed by glomerular layer interneurons^29^ and their activation can hyperpolarize and inhibit PG cells^25^,^32^. It is possible therefore that the increased glomerular odor responses seen here are due to m2R-mediated reduction of OSN GABA_B_ receptor-mediated tonic inhibition (but see also Liu *et al*.^25^) resulting in an activity independent gain increase, as reported previously with GABA_B_ blockers^40^. Similar mechanisms have been reported in other regions where mAChRs located on interneurons suppress presynaptic GABA release and lead to increased excitatory responses^39^,^41^,^42^,^43^. Future pharmacological experiments focused on AChR activation on different OB cell types are needed to elucidate the mechanisms underlying this modulation.

Overall, these findings suggest that AChR modulation of M/T cell glomerular responses results from the combination of activity-dependent nAChR suppression that affects responses in the mid- to high concentration range and non-activity dependent mAChR-mediated enhancement of response across all concentrations. When both receptors are engaged, the nicotinic effect overcomes the excitatory muscarinic effect to produce inhibition at near saturating odor responses while leaving the enhanced responsivity at lower concentrations unchanged. Thus, the direction of glomerular response modulation is dependent upon its relative control odor response amplitude and can be predicted from the baseline odor responses of a given odorant. As previous studies^4^,^22^,^27^ primarily used fixed or relatively low odor intensities, the bidirectional modulation observed here would not have been detected.

AChR modulation of OB responses has several potential consequences on odor detection, discrimination, and learning. Overall, bidirectional modulation leads to glomerular representations of individual odorants becoming more similar as concentration increases. This suggests a novel role for ACh in modulating perceptual constancy, a phenomenon in which the perception of the odorant remains stable across varying concentrations^44^,^45^.

Further, as OB AChR activation enhances sub-threshold responses, ACh release could increase odor sensitivity, i.e. lower detection thresholds. In line with this, scopolamine injections decrease peri-threshold odor detection in humans^46^. However, a study in rats reported that odor detection thresholds were not affected by OB infusion of the AChR agonist carbachol in an odor investigation/habituation task^47^, perhaps due to the opposing m- and nAChR actions of this agonist. Further, more detailed detection threshold studies are needed to fully test this hypothesis behaviorally.

Increasing glomerular responsivity could also potentially improve discrimination. Behavioral studies have demonstrated that stronger intensity odorants are more easily discriminated^44^,^48^. Similarly, decreasing responses to maximal concentrations could prevent saturation and facilitate identification and discrimination of very strong odors. This mechansim fits well with several studies demonstrating a crucial role for ACh in olfactory discrimination^19^,^21^,^36^.

ACh-driven increased glomerular responsivity could also serve to increase odor salience and facilitate olfactory learning. Studies of olfactory learning in honeybees have demonstrated that more salient, stronger odorants lead to stronger odor-driven associations^44^. As mAChR activation has been shown to play a key role in olfactory associative learning^20^,^24^, it is possible that these effects are mediated by OB mAChR-driven increases in odor salience. Future experiments aimed at directly manipulating OB mAChR activity during odor learning could further elucidate the role of mAChRs in mediating odor salience.

## Methods

### Animals and surgery

Adult male and female mice expressing the fluorescent calcium indicator protein GCaMP2 in mitral/tufted cells under the Kv3.1 potassium channel promoter were used to investigate the cholinergic modulation of glomerular odor responses^49^. Mice were anesthetized with urethane (2 mg/kg, ip). To reduce nasal secretions, mice received injections of the blood brain barrier-impermeant muscarinic receptor antagonist, methyl scopolamine^50^,^51^,^52^, at 0.05 mg/kg, i.p., a concentration that does not affect learning^24^. Mice were secured in a custom stereotaxic apparatus and the bone overlying the OBs was removed. A small opening in the dura was made to allow bath-applied drugs to penetrate the bulb. During imaging sessions, animals were freely breathing and respiratory activity was monitored with a piezoelectric device. All methods were carried out in accordance with relevant and approved guidelines and regulations. All experimental protocols were approved by the University of Tennessee Institutional Animal Care and Use Committee.

### Odorant presentation

Mono-molecular odorants (Sigma-Aldrich, USA) that activate the dorsal surface of the OBs were delivered using a flow-dilution olfactometer described previously^30^. Separate flow controllers were used to mix the odorant and clean air-flow streams at a flow rate of 0.7 L/min in order to dilute the odorants, at different concentrations ranging from 0.01% to 8% odorant. Odor pulse duration was 1 sec with an inter-stimulus interval of at least 60 sec.

### HDB stimulation

A bipolar tungsten stimulating electrode was lowered stereotaxically into the HDB (coordinates: 0.5 mm Bregma, 0.6 mm lateral, 3.5 mm deep). Electrical stimulation consisted of a 1-sec, 50 Hz train with an amplitude range of 10–150 μA delivered 0.5 sec prior to odor delivery.

### Drug application

All drugs were diluted in Ringer’s solution and bath applied to the OB surface. The anticholinesterase neostigmine bromide (10–100 μM) (Sigma-Aldrich, USA) was used to pharmacologically increase OB ACh levels. As previously reported, we found no differences in neostigmine-induced modulation within this concentration range^31^, so data from all concentrations was pooled. Scopolamine hydrobromide (100 μM) (Sigma-Aldrich, USA) or AF-DX 116 (100 μM) (Tocris, USA) was used to block to mAChRs and mecamylamine hydrochloride (100 μM) (Sigma-Aldrich, USA) was used to block nAChRs. Oxotremorine sesquifumarate salt (10 μM) (Sigma-Aldrich, USA), McN343 (100 μM) (Tocris, USA) and nicotine (10 μM) (Sigma-Aldrich, USA) were used to pharmacologically activate mAChRs or nAChRs, respectively.

### Optical imaging and analysis

Imaging was performed on a Scientifica Slicescope equipped with Olympus 4× (0.28 NA) and 10× (0.3 NA) objectives. The dorsal surface of the OBs was illuminated with a LED light source centered at 480 nm. GCaMP2 signals were band-pass filtered with a Chroma emission filter (HQ535/50) and collected using a CCD camera at 25 Hz (NeuroCCD-SM256, Redshirt Imaging). For glomerular response quantification, odor-evoked trials were corrected for photo-bleaching by subtracting a no-odor trial. After applying a low pass spatial filter (3 × 3 median), the odor-evoked change in fluorescence (ΔF) was calculated by subtracting the 5 frame average immediately preceding odor onset from the 5 frame average centered on the peak of the response generated by the first respiration. The relative change in fluorescence (ΔF/F) was calculated by dividing the odor-evoked change in fluorescence by the resting fluorescence. For analysis, individual discrete glomerular responses were visually identified and the ΔF/F response was measured at the center of each glomerulus (2 × 2 pixel average). Responsive glomeruli were defined as having a ΔF/F greater than that of the mean ± 2SD of the background fluorescence. Odorant concentrations that elicited a response below the background response were considered below threshold. At low concentrations, each odorant was presented several times and the average ΔF/F response was used for all comparisons. High concentrations (greater than 5% s.v.) were presented only once to prevent any potential short-term adaptation.

To explore the effects of HDB stimulation (HDBS) and the different drugs on the glomerular responses across the entire concentration range, only animals in which maximum odor responses were observed were used. The maximum response was determined when increasing odor concentration did not lead to a significant increase in the response amplitude. In these animals, we only selected the glomeruli that displayed responses across a large concentration range, could be fit to a sigmoid function, and in which an experimentally derived maximal response could be obtained in control condition. Glomeruli that did not show different responses to different concentrations or did not follow a sigmoid curve were excluded from this analysis. In the selected glomeruli, odor responses in control and HDBS and/or drug conditions were normalized to the maximum control odor response magnitude. Control and post-treatment (HDBS or drug) curves were then fitted using GraphPad Prism software. For EC50 and slope calculation, glomerular concentration-response curves were normalized to every condition’s maximum, in order to obtain relevant EC50 values for each condition^34^. The data were only included if both control and post-treatment curves had reliable fits to a sigmoid curve (r^2^ > 0.8).

To account for inter-glomerular differences in sensitivity, control and post-treatment normalized responses were analyzed. All the normalized responses were pooled regardless of odor or concentration and were grouped in ascending order. The normalized control responses were divided into ten, 10% amplitude intervals. Within each interval, the mean control and corresponding post treatment responses were plotted as a function of the normalized control intervals (Fig. 2E). Statistical analyses (one-way ANOVA, t-tests and Bonferroni post hoc tests) were performed using GraphPad Prism software. Data are expressed as mean ± SEM unless otherwise noted.

Principal Component Analysis (PCA) was used to compare dorsal surface odor representations at each concentration in both control and HDBS conditions. Only animals that showed a pre- and HDBS sigmoid fit to a given odorant for all their glomeruli were selected. For each animal (n = 6), the distance between the projection of the minimum and maximum concentration onto PC1 (which accounted for >68% of total variation) was compared in the pre- versus HDBS condition.

## Additional Information

**How to cite this article**: Bendahmane, M. *et al*. Increased olfactory bulb acetylcholine bi-directionally modulates glomerular odor sensitivity. *Sci. Rep*. **6**, 25808; doi: 10.1038/srep25808 (2016).
